# Analytical validation of the 7-gene biosignature for prediction of recurrence risk and radiation therapy benefit for breast ductal carcinoma *in situ*

**DOI:** 10.3389/fonc.2023.1069059

**Published:** 2023-05-19

**Authors:** David Dabbs, Karuna Mittal, Scott Heineman, Pat Whitworth, Chirag Shah, Jess Savala, Steven C. Shivers, Troy Bremer

**Affiliations:** ^1^ PreludeDx, Laguna Hills, CA, United States; ^2^ University of Tennessee, Knoxville, TN, United States; ^3^ Nashville Breast Center, Nashville, TN, United States; ^4^ Department of Radiation Oncology, Taussig Cancer Institute, Cleveland Clinic, Cleveland, OH, United States

**Keywords:** DCIS - breast ductal carcinoma *in situ*, DCIS biomarkers and morphogenetic mechanisms, radiation therapy, analytical validation, radiogenomics, immunohistochemistry, DCISionRT

## Abstract

**Purpose:**

Ductal carcinoma *in situ* (DCIS), is a noninvasive breast cancer, representing 20-25% of breast cancer diagnoses in the USA. Current treatment options for DCIS include mastectomy or breast-conserving surgery (BCS) with or without radiation therapy (RT), but optimal risk-adjusted treatment selection remains a challenge. Findings from past and recent clinical trials have failed to identify a ‘low risk’ group of patients who do not benefit significantly from RT after BCS. To address this unmet need, a DCIS biosignature, DCISionRT (PreludeDx, Laguna Hills, CA), was developed and validated in multiple cohorts. DCISionRT is a molecular assay with an algorithm reporting a recurrence risk score for patients diagnosed with DCIS intended to guide DCIS treatment. In this study, we present results from analytical validity, performance assessment, and clinical performance validation and clinical utility for the DCISionRT test comprised of multianalyte assays with algorithmic analysis.

**Methods:**

The analytical validation of each molecular assay was performed based on the Clinical and Laboratory Standards Institute (CLSI) guidelines Quality Assurance for Design Control and Implementation of Immunohistochemistry Assays and the College of American Pathologists/American Society of Clinical Oncology (CAP/ASCO) recommendations for analytic validation of immunohistochemical assays.

**Results:**

The analytic validation showed that the molecular assays that are part of DCISionRT test have high sensitivity, specificity, and accuracy/reproducibility (≥95%). The analytic precision of the molecular assays under controlled non-standard conditions had a total standard deviation of 6.6 (100-point scale), where the analytic variables (Lot, Machine, Run) each contributed <1% of the total variance. Additionally, the precision in the DCISionRT test result (DS) had a 95%CI ≤0.4 DS units under controlled non-standard conditions (Day, Lot, and Machine) for molecular assays over a wide range of clinicopathologic factor values. Clinical validation showed that the test identified 37% of patients in a low-risk group with a 10-year invasive IBR rate of ~3% and an absolute risk reduction (ARR) from RT of 1% (number needed to treat, NNT=100), while remaining patients with higher DS scores (elevated-risk) had an ARR for RT of 9% (NNT=11) and 96% clinical sensitivity for RT benefit.

**Conclusion:**

The analytical performance of the PreludeDx DCISionRT molecular assays was high in representative formalin-fixed, paraffin-embedded breast tumor specimens. The DCISionRT test has been analytically validated and has been clinically validated in multiple peer-reviewed published studies.

## 1 Introduction

The diagnosis of ductal carcinoma *in situ* (DCIS) has increased dramatically since the introduction and routine utilization of screening mammography (1). About 50,000 women are diagnosed with DCIS each year in the United States (2). Breast conservation surgery (BCS) followed by radiation therapy (RT) is the mainstay for DCIS treatment. BCS plus RT has been validated as a successful strategy to reduce the in-breast recurrence rate in patients diagnosed with DCIS in multiple RCTs (3), and BCS plus RT has been broadly adopted as a sufficient alternative approach to mastectomy (4–7), but not directly compared with mastectomy in a RCT. However, preventing over and undertreatment with radiotherapy remains the key challenge in the optimal treatment of DCIS given the heterogenous nature of the disease. Although the traditional clinicopathologic factors have been associated with risk of recurrence, they do not accurately determine an individual patient’s recurrence risk, or importantly, the clinical benefit from adjuvant radiation therapy (3, 8–15). Additionally, pooled analysis of the multiple randomized clinical trials have shown that traditional clinicopathological factors have limited capability in risk stratifying the DCIS patients and that there has not been a clinicopathologic patient subpopulation that has not benefited significantly from RT (3).

The goal of primary therapy for DCIS is to prevent invasive in-breast recurrences (IBR), as patient survival overall is excellent, with the choice of local treatment not impacting disease-specific or overall survival (16). At this time, current NCCN guidelines recommend that patients with DCIS should receive either 1) mastectomy, or 2) BCS with adjuvant RT, or 3) BCS alone without adjuvant RT (16). After definitive breast conserving surgery, 70% to 80% of women with DCIS will not have any IBR within 10 years, and 15% or fewer patients will benefit from adjuvant RT (3, 13). Thus, of the population of women treated with BCS, on an average 85% of women will not benefit from RT after BCS for preventing any IBR within 10 years. Consequently, BCS without RT is an option when the individual is considered to be “low-risk”, as for a low-risk patient, the absolute risk reduction of in-breast recurrence may not be large enough to justify the risks associated with RT (16). The definition of “low-risk” has been described in general, commonly referencing prognostic clinicopathologic factors (nuclear grade, tumor size, patient age, and margin status). Typically, young age, high nuclear grade, tumor size > 2 cm, positive or close margins, or the presence of palpability have been considered high-risk features, while “low-risk” has been defined as the absence of these high-risk clinicopathological factors, thus allowing for preferential treatment with endocrine therapy in hormone receptor-positive patients (16).

Several factors have been identified to contribute towards local recurrence and different classifiers based on clinicopathological factors have been tested with the goal of guiding patients to an appropriate level of treatment. Herein, Van Nuys Prognostic Index (VNPI) (that combines tumor size, margin size, grade, comedo- necrosis, and patient age) stratifies patients into scores ranging from 4 to 12 that lead to treatment recommendations of BCS, BCS+RT, or mastectomy (17, 18). However, the VNPI criteria does not provide a risk of recurrence after breast conserving surgery with and without radiation therapy and does not predict RT benefit (19).

Another clinicopathological factors-based nomogram combined data from seven variables (age, family history, detection method, grade, necrosis, margin size, and number of excisions), and accounts for the year of surgery and the absence of adjuvant RT and/or hormone therapy to predict the 10-year IBR rates (20). However, the nomogram specifies that all patients have an equally reduced score from radiation therapy, such that all patients are expected to benefit uniformly from RT. Despite the use of clinicopathologic driven risk stratifications, prospective trials have not demonstrated any clinicopathologic criterion which was predictive of a cohort of patients with DCIS that do not benefit with respect to local control from radiation therapy (3). Based on this, there has been no clear-cut low-risk population based on clinicopathologic features (21).

Despite the use of these “low-risk” clinicopathologic-driven risk stratifications, prospective trials have not demonstrated any clinicopathological criterion predictive of a cohort of patients with DCIS that do not benefit with respect to local control from radiation therapy (3). Based on this, there has been no clear-cut low-risk population based on clinicopathologic features (21). However, improved risk stratification of DCIS may be achieved by employing a robust biological risk assessment, integrating molecular biology with clinical and pathologic factors as suggested by the NCI to provide an integrated assessment of ipsilateral recurrence risk as the basis to guide therapy and help prevent overtreatment and undertreatment (22).

Multiple studies have identified a number of molecular biomarkers that are prognostic for DCIS local recurrence rate including the status of HER-2 amplification (23), negative hormone receptor status (24, 25), and immunohistochemical detection of a range of biomarkers, including COX2 (26, 27), Ki67 (27), p16 (26–28), p53 (29, 30), p21 (31), and BNIP3 (32). But none of these markers have individually addressed the question of the expected differential benefit from radiation therapy. Thus, the utility of these biomarkers was limited and not clinically adopted for risk stratification of DCIS patients. Moreover, a commercialized RNA based 12 gene DCIS test, Oncotype DX DCIS Score^®^, was validated retrospectively using a low-risk EORTC clinical trial population and an observational Canadian population (33, 34). The test provides an estimated risk of 10-year total and invasive recurrence risk but does not report any information on the predicted benefit of radiation therapy. Validation studies demonstrated that the test was prognostic but that 10-year IBR risks were higher for the intermediate than the high DCIS Score groups (10-year IBR intermediate DCIS score group 33% *vs.* 10-year IBR High DCIS score group 27.8%) (34–36) and there was no direct interaction of the DCIS score with RT in the study (35). Of note, the low risk and high risk groups had approximately the same relative benefit from RT (19, 33).

The DCISionRT test is a biosignature that was developed to address this unmet need and is the first genomic test to predict radiation therapy benefit in patients with DCIS. DCISionRT provides a comprehensive assessment of the woman’s ipsilateral breast cancer recurrence risk after breast-conserving surgery with and without RT by integrating tumor molecular biology and clinicopathology. The biosignature/test is a multianalyte assay with algorithmic analysis (MAAA). The test integrates protein expression of seven critical genes measured in formalin-fixed paraffin-embedded (FFPE) tumor tissue samples with four clinicopathologic factors (age, tumor extent, tumor palpability, tumor margin status) as inputs to the proprietary algorithm to report a score (37–41).

The DCISionRT test was derived from research performed at UCSF. The investigators identified that the level of P16^INK4A^ in combination with KI67 provided a significant assessment of subsequent invasive breast event risk. In a subsequent nested case-control study that analyzed a DCIS cohort treated with BCS without RT, invasive-IBR was associated with palpability, young age, and the expression of p16, COX-2, and Ki-67 (27).

Following the discovery research at UCSF, PreludeDx (Laguna Hills, CA) further developed the test to account for the interactions between the different biomarkers and the clinicopathology factors, employing machine learning. A nonlinear algorithm was developed such that the value of a given risk factor depends on the values of other risk factors (37). This enabled the DCIS biosignature to account for the interdependencies and activation of the oncogenic pathways commonly dysregulated in DCIS such as estrogen response pathway, HER2 pathway, cell cycle, survival and stress response leading to increased proliferation and cell survival. The biosignature was parameterized and tested using multiple cross-validation and produced a continuous score. Parameterization was performed with the training folds and evaluated using the validation folds of the UUH/UMASS study cohort (37). A continuous score ranging from zero to ten, termed the Decision Score (DS), was reported for each patient as the median of the multiple cross-validated results (37).

Furthermore, the results from initial studies using the DCISionRT biosignature showed there was a subset of the patients with high DS scores who had higher risk of recurrence after BCS plus RT treatment. Given the heterogenous nature of the disease it was hypothesized that the biology underlying these high-risk patients was different than other patients and some specific pathway(s) were driving the aggressiveness and residual risk after BCS plus RT. Interestingly it was observed that a significant percentage of this high risk population was HER2 positive, further validating the findings from previous studies that DCIS shares similar genomic heterogeneity to invasive breast cancer comprising lesions that vary in their clinical presentation and outcomes. Thus, in order to further identify the subset of the patients with a greater risk of recurrence after BCS plus RT, additional pathways regulated by the existing DCISionRT biomarkers that could impact progression of breast cancer and contribute to the resistance of standard therapies were investigated, and the K-RAS pathway was identified as the putative pathway contributing to the aggressiveness in the high risk group. An algorithm was pre-specified to combine biomarkers (used by the DS biosignature) in a novel manner based on the biologic hypothesis that an activated K-RAS pathway would drive a proliferative, aggressive disease profile and thus could identify a subgroup of patients with higher residual risk after adjuvant RT i.e., a Residual Risk Subtype (RRt) (38).

The DCISionRT test has been validated to be prognostic for IBR risk and predictive for RT benefit in multiple clinical validation studies in over 1,600 patients from five different cohorts with long-term outcomes (37, 38, 40–42). In a prospective clinical utility study, RT recommendations were changed for about 40% of DCIS patients when DCISionRT was incorporated into routine treatment decision management, identifying low-risk patients who may avoid unnecessary and costly RT and associated potential toxicities (avoiding overtreatment), as well as identifying higher risk patients to appropriately receive a necessary, beneficial treatment (avoiding undertreatment) (39). In addition, a cost-effective analysis for use of DCISionRT in patients undergoing BCS for DCIS with or without RT has shown that treating the Elevated Risk group patients based on DCISionRT was most cost effective when compared to treating all patients diagnosed with DCIS (43).

To be clinically applicable, a test to guide DCIS treatment strategy must have validated analytical performance for the molecular assays and validated clinical performance for the reported test results. The clinical and analytic validity of a multianalyte molecular assay relies on the analytes, reagents, precise experimental techniques, correct application of controls, and ultimately, an accurate interpretation of the data based on all the aforementioned factors in the post-analytic phase. Here we report the analytic performance of the DCISionRT test using well-established methods as per the recommended guidelines. The analytic performance of the DCISionRT assay system was performed at the PreludeDx centralized clinical laboratory, including all steps involved in clinical lab implementation. The clinical performance and clinical utility of the DCISionRT test was also summarized with clinical metrics and performance statistics.

## 2 Materials and methods

### 2.1 Study design

The analytical validation of each molecular assay was based on the Clinical and Laboratory Standards Institute (CLSI) guidelines for Quality Assurance for Design Control and Implementation of Immunohistochemistry Assays (44) and College of American Pathologists/American Society of Clinical Oncology (CAP/ASCO) recommendations for analytic validation of Immunohistochemical assays (45). Analytical validation demonstrates the assay’s ability to measure the analyte of interest in specimens representative of the population of interest in the clinical laboratory. The molecular assay analytic sensitivity, analytic specificity, and analytic accuracy were determined by comparing observed results in representative tissues with known or expected positive and negative expression. Intra-laboratory analytic precision for each molecular assay was determined as the extent of agreement among results obtained by replicate testing of representative tissue specimens under specified variable assay conditions, including different equipment, antibody lots and testing day assessed by two pathologists, while reproducibility was assessed as the percent agreement between different pathologists (45). In-line with the goal of improving DCIS treatment management by ruling out overtreatment with RT, the clinical performance of the test for RT benefit was also reported as summary statistics based on standard definitions (46).

### 2.2 Analytical validation performance

The protein expression of each gene was assayed using immunohistochemistry in accordance with laboratory Standard Operating Procedures (SOPs). The primary monoclonal antibodies in each of the molecular assays were individually validated with the same SOPs, using cell lines and tissues that were fixed in 10% neutral buffered formalin and paraffin-embedded (FFPE) per CAP/ASCO guidelines (45). The tissue samples were assayed on a Leica BOND III, using monoclonal antibodies with pre-specified titers, a Leica Diluent dilution buffer, and specified pre-treatment and IHC protocols (36, 47). All biomarkers were scored for intensity and percentage (H-score) by board-certified pathologists at the PreludeDx CLIA laboratory. External negative isotype controls for each protein biomarker consisted of tissue samples that were processed with an isotype antibody with the same concentration and assay conditions as the test samples. Cell lines were selected with a known positive expression for each protein biomarker, and in addition, the HER2 cell lines were selected to have known (negative, 1+, 2+, or 3+) expression (48). For each protein biomarker, tissue control samples with expected negative or positive expression were assayed to confirm that the observed expression was consistent with the expected expression. Normal organ (n=25) tissue samples (n=100) in a tissue microarray (TMA, BIOMAX FDA9ww2) were characterized using the molecular assays and reported in **Supplemental Table 2**.

### 2.3 Molecular assay sensitivity, specificity, and accuracy

Analytic sensitivity and analytic specificity for each molecular assay were estimated as positive and negative concordance of the observed results, respectively, with either expected expression for a given tissue type or observed results from a standard referent inter-lab assay for comparison. Accuracy for each molecular assay was estimated as overall concordance of the observed results with results from a referent inter-lab assay (44). The molecular assays were read in a post-analytic phase by board-certified pathologists at the PreludeDx CLIA laboratory or a reference laboratory. A mix of invasive carcinomas, ductal carcinoma *in situ* tumor (DCIS), and normal organ tissue samples were purchased as tissue micro arrays (Biomax). Annual proficiency testing results (CAP) were utilized to augment the analytic validity of molecular assays for specific markers (PR, HER2, P16). The reported assay results for biomarkers PR, KI67, P16, and SIAH2 were summarized as percentages, while HER2 was summarized per CAP/ASCO adapted for DCIS (49), FOXA1 was summarized as a total H-score, and COX2 was summarized as an Allred Score (49). An inter-lab comparison was done for the PgR, Her2 and KI67 for total concordance as these assays were readily performed at other laboratories.

### 2.4 Molecular assay precision

The intra-lab molecular assay precision was determined by assaying consecutive sections of multiple tissue samples in a constructed tissue micro array over multiple days, using different primary antibody lots and equipment. The DCIS cases selected were each from a unique DCIS patient and were DCIS tumors with no microinvasion. Specifically, fifteen (15) tissue samples were constructed in a tissue micro array and assayed at PreludeDx (CLIA/CAP) in accordance with validated lab SOPs for controlled non-standard conditions (machine n=2, antibody lot n=2, and day of run n=3). At the beginning of a consecutive 5-day validation period, 12 TMA consecutive sections were cut, slide mounted, and stored at room temperature. Two pathologists independently scored the processed tissue samples in the post-analytical phase. Each molecular assay was normalized to a 100-point scale and the dispersion of the mean was calculated by comparing each of the replicate sample average scores from two pathologists to the mean score of the set of replicate samples, which was reported as the % standard deviation for ease of comparison. CV was also reported as % std/mean score. The variance in the result attributed to each of the analytic factors combined within- and between-run random error was assessed using mixed effects modeling of the following form that was applied to assess the differences between mean *vs*. replicate biomarker score results using equation 1.

**Equation 1:**

*Score_ij_* = SCORE _*ij*_ + Run_*ij*_ + Lot_*ij*_ + Machine_*ij*_ + ϵ_*ij*_

where *i* is the sample (1-15), and *j* is the reproducibility state (Run=3, Lot=2, Machine=2); *Score* is the individual replicate scores from each biomarker assay, SCORE is the mean score for each biomarker assay averaged over j reproducability states, and ϵ_*ij*_ ∼ *iid N* (0, σ^2^_ε_) is (independent and identically distributed) random error.

The mixed effect modeling was implemented with the lmer function from the lme4 package in R version 4.1.1. Summary statistics were reported as percent total variance for reproducibility variables (Run, Lot, Machine), standard deviation, and percent of total variance.

### 2.5 Multianalyte assay with algorithm analysis: reproducibility

In addition to the pre-specified primary aim to analytically validate the multianalyte assay, the reproducibility of the reported MAAA results was determined, with an aim of highly reproducible test results with a total standard deviation (SD) of less than 0.4 DS units. Specifically, the seven biomarker results for each of the replicate DCIS TMA samples assayed with controlled non-standard conditions (machine, antibody lot, and run) were combined with a set of pre-specified clinicopathologic factors using SOPs. The clinicopathologic factors were age (40, 55, or 70 years; representing young pre-menopause, perimenopause, and older post-menopause), extent (5 mm, 15 mm and 45 mm; representing small, medium and larger DCIS), margin status (negative or positive ink on tumor), and palpability (no, yes). The DCISionRT algorithm was used to calculate the Decision Score (DS) for varying clinicopathologic factors (n=36) and seven biomarkers for the DCIS tissue samples (n=15), which yielded 540 mean DS results for each of the 12 variable assay conditions (machine n=2, antibody lot n=2, and day of run n=3). The variance in the DS result attributed to each of the analytical factors combined within- and between-run random error was assessed using a mixed effects modeling of the following form that was applied to the assess the differences between 540 mean *vs.* 6,480 individual DS results using equation 2.

**Equation 2:**


DSijk=DSijk+Runijk+Lotijk+Machineijk+ϵijk

Mixed effect modeling of precision variance, where i is the sample (1-15), j is the unique clinicopathologic set (1-12), and k is the reproducibility state (Run=3, Lot=2, Machine=2); *DS* are the average test results from two independent pathologist assessments, DS is the mean DS test result averaged over k reproducibility states, and ε_*ijk*_ ∼ *iid N*(0,σ^2^_ε_) is random error.

The mixed effect modeling was implemented with the lmer function from the lme4 package in R version 4.1.1. Summary statistics were reported as percent total variance for reproducibility variables (Run, Lot, Machine), standard deviation, percent of total variance, and 95% confidence interval of DS results.

### 2.6 Ethics approvals

Clinical performance of DCISionRT was summarized from previous studies approved by local ethics committees/boards from Uppsala University Regional Ethical Review Board (37), University of Massachusetts Medical School Tissue and Tumor Bank Institutional Review Board (37), the Kaiser Permanente Northwest Institutional Review Board (41), Umeå University Ethics Review Committee (40), and the Royal Melbourne Hospital and Affiliated Hospitals Ethics Review Board (42), as previously published.

### 2.7 Clinical performance: multianalyte assay with algorithm analysis

The clinical performance of the DCISionRT test for RT benefit was summarized for all validation studies by biosignature risk groups as absolute 10-year IBR rates after BCS treatment with and without RT, absolute risk reductions (ARR), and the number of patients needed to treat (NNT) (37, 38, 40, 41). NNT was defined as 1/ARR. Summary performance statistics (NPV, PPV, sensitivity, specificity) were calculated based on a confusion matrix and standard definitions for count data (equation 3 presented in **Supplementary Table 4**) adapted to right-censored event data (equation 4) (46).

**Equation 4:**

**Table:** 

	RT Benefit	No RT Benefit	Summary Statistics
**Test Positive for** **RT Benefit (X>z), N_POS_**	a = $\hat{TP}$ $\hat{=\text{ARR}}$ _KM_(t|X>z) ^*^ N_POS_	b = $\hat{FP}$ = N_POS_ – a	**PPV** = a/(a+b) = $\hat{ARR}$ _KM_ (t|X>z); N_POS_ = a+b
**Test Negative for RT Benefit (X≤z), N_NEG_**	c = $\hat{FN}$ = N_NEG - d_	d = $\hat{TN}$ = =(1- $\hat{ARR}$ _KM_(t|X≤z)) *N_NEG_	**NPV** = d/(c+d) = 1- $\hat{ARR}$ _KM_(t|X≤z); N_NEG_ = c+d
**Summary Statistics**	**Sensitivity** = a/(a+c) ^∗^ 100	**Specificity** = d/(b+d) ^∗^ 100	${\hat{ARR}}_{KM}(\text{t}|\text{X}>\text{z})=$ ${\hat{S}}_{\text{KM}}\left(\text{t}|\text{X}>\text{z},\text{No RT}\right)\;-$ ${\hat{S}}_{\text{KM}}\left(\text{t}|\text{X}>\text{z},\text{ RT}\right)$

Summary statistics using right censored event data. NPV = negative predictive value; PPV = positive predictive value; $\hat{TP}$ = estimated true positive; $\hat{TN}$ = estimated true negative; $\hat{FP}$ = estimated false positive; $\hat{FN}$ = estimated false negative; $\hat{ARR}$ (t) = estimated absolute IBR risk reduction (ARR) from RT by Kaplan-Meier analysis at time t; $\hat{S}$ _KM_ (t|X>z, RT) = Kaplan Meier IBR rate estimate at time t evaluated for Test Positive group (X>z) treated with RT; $\hat{S}$ _KM_(t|X>z, No RT) = Kaplan Meier IBR rate estimate at time t evaluated for Test Positive group (X>z) treated with No RT; $\hat{ARR}$ (t|X>z) = Estimated ARR by Kaplan Meier analysis at time t evaluated for Test Positive Group (X>z); $\hat{ARR}$ (t|X≤z) = Estimated Absolute Risk Reduction at time t evaluated for Test Negative Group (X≤z); X = Test covariate; z = Test covariate threshold; N_POS_ = Number of Patients with Test Positive; N_NEG_ = Number of Patients with Test Negative.

## 3 Results

### 3.1 Molecular assay characterization

The molecular assays utilized a selected primary antibody for each biomarker, as characterized in **Table 1** (Manufacturer, Clone Type/Host, Isotype, and Immunogen). Isotype responses for all antibodies were negative (0+ 100%) for known positive tissue controls for each antibody. Representative results for organ tissue positive controls are illustrated in **Supplemental Figure 1**. Detection kit (antibody-negative) controls were negative (0+ 100%) in the consecutive sections of the known positive tissue controls for each antibody in **Supplemental Figure 1.**

**Table 1:** Antibody characteristics.

Protein	Manufacturer	Clone	Type/Host	Isotype	Immunogen
PgR	Leica	16	Monoclonal Mouse	IgG1	N-Terminal Region of PgR form A
HER2	Cell Marque	SP3	Monoclonal Rabbit	IgG1	Positions 654 and 655 of isoform a, positions 624 and 625 of isoform b
Ki-67	Dako	MIB-1	Monoclonal Mouse	IgG1 kappa	cDNA 1002bp fragment
COX-2	Cell Marque	SP21	Monoclonal Rabbit	IgG	A synthetic peptide from the C-terminus of rat cox2
FOXA1	Cell Marque	2F83	Monoclonal Mouse	IgG1 kappa	Recombinant human GST-FOXA1 protein encompassing amino acids 7-86.
p16/INK4A	Ventana	E6H4	Monoclonal Mouse	IgG_2a_	Recombinant protein corresponding to full length p16.
SIAH2	Cell Marque	HC/LC C39S	Monoclonal Mouse	IgG_2a_	Synthetic peptide corresponding to a region near the N-terminus of SIAH

The cell lines were processed and embedded in paraffin similar to the tissue samples. Observed results for each cell line and FFPE tissue control were concordant with previously reported expression profiles (expected results) and are presented in **Table 2**. Representative molecular assay results in DCIS tissue samples with varying protein expression are shown in **Supplemental Figure 2**. Summary results for positive or negative expression in normal organ tissues are listed in **Supplemental Table 1**.

**Table 2:** Observed and expected molecular assay results in cell lines and tissue controls.

Gene	Cell Line	Expected Expression	Observed Expression	Tissue Control	Expected Expression	Observed Expression
PgR	MCF-7 (50)	Positive	3+ 50% 2+ 25% 1+ 15%	Tonsil Breast CA	Negative Strong	0 100% 3+ 95%
HER2	SKB-R3 (51) MDM-MB-453 (52) MDM-MB-175 (53) MDM-MB-231 (51)	3+ 2+ 1+ 0	100% 3+ 99% 2+ 100% 1+ 99% 0	Tonsil Breast CA	Negative Strong	0 100% 3+ 100%
Ki-67	RAMOS	Positive	3+ 80% 2+ 15% 1+ 5%	Cerebrum Tonsil mantle zone (MZ)	Negative Strong	0 100% 3+ 5%, 2+ 15%, 1+ 30%, 0 50%
COX-2	COLO-205 (54)	Positive	3+ 75% 2+ 20% 1+5%	Uterus Liver cirrhosis	Negative Strong	0 100% 3+ 10%, 2+ 85%, 1+ 5%, 0 0%
FOXA1	MFC7 (55)	Positive	3+ 20% 2+ 50% 1+ 25%	Normal uterus endometrium Prostate adenocarcinoma	Negative Strong	0 100% 3+ 80%, 2+ 10%, 1+ 5%, 0 5%
p16/INK4A	Hela (56)	Positive	3+ 95% 2+ 5% 1+ 0%	HPV-negative ovarian adenocarcinoma HPV-positive squamous CA lymphoid tissue	Negative Strong	0 100% 3+ 30%, 2+ 50% 1+ 10%, 0 5%
SIAH2	RAMOS	Positive	3+ 85% 2+ 10% 1+ 5%	Cerebrum Lung carcinoma	Negative Strong	0 100% 3+ 40%, 2+ 25%, 1+ 5%, 0 30%

The positive percent concordance (estimating analytic sensitivity) and negative percent concordance (estimating analytic specificity) of the molecular assays with expected results are summarized in **Table 3** for specified thresholds for each biomarker. The tissue type used to validate each biomarker is summarized in **Table 3** and detailed in **Supplemental Table 2**.

**Table 3:** Positive concordance (sensitivity), negative concordance (specificity), and total inter-lab concordance (accuracy/reproducibility).

Biomarker	PgR	HER2	p16/INK4	Ki-67	COX2	SIAH-2	FOXA1
Specificity	84/88 (95%)	172/172 (100%)	108/109 (99%)	56/56 (100%)	120/122 (98%)	53/53 (100%)	71/71 (100%)
Negative threshold	0-1%	0+ or 1+	0	<5%	Allred 0	0 (H Score)	<25 (H Score)
Tissue Types	Normal organ panel	Normal organ panel	Normal organ panel	Normal organ panel	Normal skin and uterus endometrium	Normal cerebrum and adrenal gland	Normal organ panel
Sensitivity	84/88 (95%)	89/89 (100%)	49/50 (98%)	49/49 (100%)	101/106 (95%)	113/116 (97%)	86/90 (96%)
Positive threshold	>5%	3+	>1%	≥15%	Allred ≥4	≥10 (H Score)	>50 (H Score)
Tissue Types	Invasive breast adeno-carcinoma	Invasive breast adeno-carcinoma	Head and neck and cervix	Invasive breast adeno-carcinoma	Liver, DCIS and invasive breast carcinoma	Colon adeno-carcinoma and lung carcinoma	Prostate adenocarcinoma and colon adenocarcinoma
Total Percent Concordance	74/74 (100%)	215/215 (100%)	48/50 (96%)	105/105 (100%)	Not Available	Not Available	Not Available

Tissues with expected negative and positive expression for each biomarker were a major component of validating molecular assays, including the use of breast cancer tumors, other tumors, and normal organ tissue samples. For the tissue samples that were expected to be positive and negative, the corresponding percent positive concordance (estimating analytic sensitivity) and percent negative concordance (estimating analytic specificity) were ≥95% for all of the molecular assays. The total concordance (estimating accuracy/reproducibility) was ≥95% for all of the molecular assays with inter-lab comparisons available.

The precision of each molecular assay under controlled non-standard conditions is summarized in **Table 4**, accounting for differing antibody lots, machines, and runs on non-consecutive days. The overall standard deviation of the dispersion of the mean over replicates under controlled non-standard conditions was 6.6%, and the analytic variables Lot, Machine and Run accounted for (<1%) of the total variance on average.

**Table 4:** Molecular assay precision: mixed effect modeling.

Molecular Assay	HER2	P16	SIAH2	KI67	FOXA1	COX2	PR	OVERALL
Score Mean	33	10	22	21	92	40	30	38
Score Range (min, max)	0,100	0,100	1,80	3,80	33,100	0,100	0,100	0,100
RUN std (%var)	0.1 (<1%)	0.4 (14%)	<0.1 (<1%)	2.2 (15%)	<0.1 (<1%)	3.0 (5%)	0.9 (4%)	<0.1 (<1%)
LOT std (%var)	0.7 (<1%)	0.2 (3%)	1.4 (9%)	<0.1 (0%)	<0.1 (<1%)	0.5 (<1%)	<0.1 (<1%)	<0.1 (<1%)
MACHINE std (%var)	<0.1 (<1%)	0.3 (11%)	<0.1 (<1%)	0.3 (<1%)	<0.1 (<1%)	0.5 (<1%)	0.02 (<1%)	<0.1 (<1%)
Standard deviation (100-point scale)	7.5	1.0	4.6	5.6	1.7	12.9	4.2	6.6

The reproducibility of the DCISionRT assay system was assessed as the dispersion of the DS mean, under controlled non-standard conditions (antibody lot, machines, and runs on non-consecutive days) in addition to implicit within and between run variances. The analysis of the sources of variance of the dispersion of DS from the DS mean is shown in **Table 5**. There were 540 mean DS test results that were derived from the molecular assay results for the seven (7) biomarkers and the 15 unique DCIS tumor tissue samples combined with the clinicopathologic factor sets. The reported DS results ranged from 0.8 to 10 with a mean of 5.7 (1^st^ quartile: 2.8, 3^rd^ quartile 9.2). The overall standard deviation of the dispersion of the mean over replicates under controlled non-standard conditions was low (0.20/10 point scale), and the analytic variables Lot, Machine and Run accounted for (<1%) of the total variance on average, where the DS confidence interval was (95%CI: -0.4, 0.4) on a 10-point scale.

**Table 5:** Multianalyte assay with algorithm analysis reproducibility: mixed effect modeling.

	Reproducibility Variables			
Analytic Variable	Reagent Lot (2)	Run (3)	Machine (2)	Within-run
DS std, (% var) (% of variance)	0.005 (0.8%)	0.018 (0.05%)	0.013 (0.4%)	0.20 (99%)

DS = DS.mean + (1 | MACHINE) + (1 | LOT) + (1 | RUN), n=6480.

### 3.2 Clinical performance

The absolute risk reduction (ARR) from RT in 10-year IBR rates are reported for DCISionRT Risk groups for the SweDCIS randomized clinical trial cohort and an observational cohort combined from Upsala University Hospital, Sweden, and the University of Massachusetts, Kaiser Permanente Northwest, Royal Melbourne, Australia studies (38) in **Table 6A**. In the SweDCIS RCT validation cohort, 47% of patients (n=240/504) were classified into the DCISionRT Low Risk group and there was a non-significant difference in the 10-year invasive IBR rates for with versus without RT, with an average difference of 1%, while the remaining patients with elevated DS results (n=264/504) had a 9% ARR in the 10-year invasive IBR rate. In the combined observational cohort (38), there were 37% (338 of 926) of patients categorized into the DCISionRT Low Risk group that had a non-significant 1% difference in the 10-year invasive IBR rate for women treated with versus without RT, while the remaining patients with elevated DS results (n=588/926) had a 9% ARR in the 10-year invasive IBR rate. In both of these validation cohorts, the number of patients needed to treat (NNT) to prevent one invasive IBR within 10 years was about 100 for those in the Low Risk group and about 11 for the remaining patients with elevated DS results.

**Table 6:** Absolute risk reduction (ARR) by risk group and study.

A. DCISionRT	Low Risk			Elevated Risk		
Study	N (%)	10-yr Invasive ARR (95%CI)	10-yr Total ARR (95%CI)	N (%)	10-yr Invasive ARR (95%CI)	10-yr Total ARR (95%CI)
Randomized Validation (SweDCIS), Cancers 2021, n=504 (40)	240 (48%)	1% (−6% to 8%)	6% (−1% to 12%)	264 (52%)	9% (2% to 17%)	16% (6% to 25%)
Modern observational validation, combined cohort, IJROBP 2022, n=926 (38)	338 (37%)	1% (−5% to 6%)	1% (−4% to 5%)	588 (63%)	9% (2% to 16%)	18% (9% to 26%)
Number Needed to Treat (NNT), modern observ-ational validation, IJROBP 2022, n=926		100	100		11	6
B. Nuclear Grade	Nuclear Grade 1		Nuclear Grade 2		Nuclear Grade 3	
Study	n (%)	Total ARR	N (%)	Total ARR	N (%)	Total ARR
EBCTCG analysis, combined four RCTs, JNCI monographs 2010, n=1614 ^3^	634 (39%)	16% (9% - 23%)	343 (21%)	14% (4%-25%)	640 (40%)	16% (8% - 23%)
Number Needed to Treat (NNT), EBCTCG 2010, n=1614 ^3^		6		7		6

The ARR in 10-year IBR rate and the corresponding NNT were also summarized by nuclear grade in **Table 6B** for patients combined from four DCIS randomized clinical trials by EBCTCG (n=1617) (3). In the overall EBCTCG combined cohort, the DCIS to invasive IBR events was about 1:1 over 10 years. For patients with nuclear grade 1 or 2 DCIS (n=977 of 1617) the ARR in 10-year total IBR rate was 15%, and for patients with nuclear grade 3 disease (n=640 of 1617) the ARR in 10-year total IBR rate was 16%. Based on an equal ratio of DCIS to invasive IBR events, the ARR in 10-year invasive IBR rate is 7.5% for nuclear grade 1 or 2 DCIS, and 8% for nuclear grade 3 DCIS. The corresponding NNT to prevent one invasive breast event in 10 years was 13 for nuclear grade 1 or 2 DCIS and 13 for nuclear grade 3 DCIS.

The clinical performance statistics negative predictive value (NPV) and positive predictive value (PPV) for 10-year RT benefit, which are corollaries to the ARR in 10-year IBR, are summarized along with sensitivity and specificity for 10-year RT benefit in **Supplemental Table 3A** for the combined observational cohort (n=926) (38) using Equation 2. The proportion of patients who benefited from RT detected by DCISionRT elevated DS results, termed clinical sensitivity for RT benefit, was 93% for invasive IBR and 96% for total IBR. In contrast, the clinical sensitivity for RT benefit for 10-year IBR based on nuclear grade 3 *vs*. nuclear grade 1 or 2 DCIS was 38% for invasive IBR and 40% for total IBR for the combined EBCTCG randomized clinical trial cohorts for RT (n=1617) (3), **Supplemental Table 3B**. The proportion of patients not benefiting from RT detected by the DCISionRT Low Risk group (termed clinical specificity for RT benefit, was 38% for invasive IBR and 41% for total IBR in the combined observational cohort (n=926) (38), whereas for nuclear grade 1 or 2 DCIS in the combined EBCTCG randomized clinical trial cohorts for RT, the clinical specificity for RT benefit was 60% for invasive IBR and 61% for total IBR (3), **Supplemental Table 3B.**

## 4 Discussion

The DCISionRT test is a 7-gene predictive biosignature comprised of multiple molecular assays for protein expression that are algorithmically combined with four clinicopathologic factors to report a continuous DS result and categorical risk groups, with corresponding 10-year total and invasive IBR rates for patients treated with BCS either with or without RT. The DCISionRT test has been previously validated in multiple observational cohort studies and the SweDCIS randomized clinical trial cohort with a prospective-retrospective study design and determined to be prognostic for 10-year IBR risk and predictive for RT benefit (37, 38, 40, 41). The DCISionRT molecular assays provided robust analytic performance for assessing protein expression of the 7 genes in the centralized clinical lab setting. The molecular assays demonstrated high analytic positive concordance rates (analytic sensitivity), negative concordance rates (analytic specificity) and total concordance (analytic accuracy/reproducibility) for the protein expression of 7 target genes.

The molecular assays demonstrated high analytic precision, with very low variation (<1%) due to controlled non-standard conditions (Run, Antibody Lot, and Machine). The reproducibility of the DCISionRT test system was high with a 95% confidence interval of less than 0.4 DS units on a scale of 0-10 (4%), accounting for varying clinicopathologic factor combinations for replicate molecular assays results obtained under controlled non-standard conditions with two independent pathology assessments of each biomarker. In clinical practice, the SOP requires that each of the independent assessments are used to provide preliminary independent DS assessments. If the two preliminary independent DS assessments differ by more than 0.5 units, then the DAP quality control algorithm automatically identifies the biomarker(s) resulting in the difference and requires a consensus score for those biomarker(s). The updated protein expression profile with consensus results is used to finalize and report the test result to ensure a high level of reproducibility case by case.

Findings from prior RCTs and more recent studies for BCS and RT have demonstrated that patients who had BCS without RT had a 10-year overall IBR rate of ~30% (DCIS plus invasive) and ~15% IBR rate for invasive breast cancer (3). The addition of adjuvant RT to BCS (BCS plus RT) reduced recurrence risk by half, with approximately 8% of patients having invasive breast cancer and the other half having DCIS within 10 years. Thus, 85% or more of patients are not expected to benefit from RT to prevent a subsequent 10-year IBR (either DCIS or invasive) within 10 years. Based on multiple validation studies, the DCISionRT Low Risk group identifies about half (40%) of the 85% of patients not expected to benefit from RT for preventing a subsequent 10-year IBR. The Low Risk group identified by DCISionRT had low 10-year IBR recurrence rates and very low ARR (non-significant 1% absolute difference with or without RT) in 10-year invasive IBR (37, 38, 40, 41). In the Low Risk group, the corresponding NNT to prevent one 10-year invasive IBR was high (about 100, **Table 6**), and equivalently, the NPV for RT benefit in 10-year IBR was high for the Low Risk group (99%). As an alternative means to assess a prognostic or predictive test, the clinical sensitivity was high for RT benefit in 10-year IBR (94% for invasive IBR and 96% for total IBR), and the clinical specificity was 38% to 40% for RT benefit. This indicates that the percentage of false negative results from the Low Risk group is quite low and that about 40% of the patients who are expected to not benefit from RT are identified by the Low Risk group. Of note, a test with high sensitivity for a treatment benefit allows patients who will be unlikely to benefit from a treatment to be safely identified. The validated low IBR rate without RT and low ARR in 10-year IBR from RT indicates that the test identifies a low-risk population of patients consistent with NCCN guidelines, who may be considered for de-escalation of RT.

Based on multiple DCISionRT validation studies with patients from four observational and one randomized clinical trial cohorts, patients with higher DS results had elevated 10-year IBR recurrence rates and clinically significant ARR for RT in 10-year IBR (37, 38, 40, 41). The NNT was between 6 and 11 for 10-year total and invasive IBR for those patients with elevated DS indicating that a limited number of these patients would need to be treated to prevent a subsequent breast cancer recurrence.

As with adjuvant RT, not all patients are expected to benefit equally from adjuvant endocrine therapy (ET). The benefit from ET may differ due to patient compliance with ET, and the risk of recurrence after BCS alone and the relative risk reduction obtained from ET may also vary with tumor molecular biology. The DCISionRT test provides 10-year risk estimates for patients treated with BCS alone, which may help in shared decision making for DCIS treatment management. Furthermore, in multivariable analysis of DCISionRT Risk groups, clinico- pathlogic factors, and treatment with RT and ET, patients treated with ET had a significantly lower IBR rate in multivariable analysis (HR=0.55, p=0.033) (38). In univariate analyses for ET benefit within DCISionRT Risk groups, only patients in the Elevated Risk group had a significantly lower IBR rate for patients treated with ET versus without ET (HR=0.34, p=0.02) (57). Thus, patients with elevated DS results may be expected to have greater absolute risk reductions from ET.

The multivariable analysis also showed that while DCISionRT risk groups contributed significantly to IBR rate, tumor grade and size, and patient age did not have a statistically significant association with IBR rate. Of these factors, nuclear grade is not a clinicopathologic factor in the DCISionRT test, but is a factor commonly used clinically to identify patients expected to benefit from radiation therapy. The analysis of the four randomized DCIS clinical trials for radiation therapy post lumpectomy identified a large number (n=1617) of patients with known nuclear grade. For patients with nuclear grade 1 or 2 in the RCTs, the ARR for RT was clinically relevant (15% 10-year IBR) and the corresponding NNT was low. Likewise, for patients with nuclear grade 3, the ARR for RT was clinically relevant (16% 10-year IBR) and the corresponding NNT was low. The results demonstrated that regardless of nuclear grade, radiotherapy was effective in reducing the absolute 10-year risk of any ipsilateral breast event. The performance metrics for nuclear grade from the four RCTs indicated that the NPV was moderate (85%), and the corresponding sensitivity (40%) and specificity (61%) were also moderate for identifying patients who would benefit from radiation therapy. This indicates that patients with nuclear grade 3 as well as those with nuclear grade 1 and 2 are expected to benefit from radiotherapy, consistent with the conclusions of the studies based on standardly reported clinical results. In addition, there are limitations in the accuracy of nuclear grade for DCIS assessed between different sites (58, 59), further limiting the utility of nuclear grade to identify patients with low-risk DCIS.

Prognostic and predictive tests are also evaluated by how they impact clinical practice. The clinical utility of the DCISionRT biosignature was reflected in the change in RT treatment recommendation observed in the prospective clinical utility study (PREDICT) with the incorporation of the DCISionRT test into routine clinical practice. In the PREDICT study, the utilization of the DCISionRT test led to a 40% change in recommendation for radiation therapy post lumpectomy for patients diagnosed with DCIS. Logistic regression analysis of the RT recommendation after DCISionRT testing indicated that the DCISionRT test result had the greatest impact on the RT recommendation, compared to clinicopathologic risk factors, physician specialty, treatment center type, patient preference and patient race (39). The recommendation for treatment with DCISionRT varied with continuous DS. In particular, those with a low DS result (DS<2) were recommended RT less often (26%), about 50% of those with a DS result between 2 and 4 were recommended RT, while RT was recommended for 95% of those with higher DS results (DS>4). In summary, the analytic performance, along with clinical validation and clinical utility studies support the continued clinical adoption of the test to guide shared decision making for DCIS treatment management. The analytic validation indicates the individual biomarkers have high performance and reproducibility and minimal variability due to standard imprecision conditions, resulting in high reproducibility of the DCISionRT test result. The test has been validated to identify patients with low recurrence risk and minimal to no absolute risk reduction benefit from RT, where 99% of the patients in the low-risk group did not benefit from RT. These patients may be good candidates for treatment with BCS without RT, depending on risk tolerance and other factors specific to the individual patient. In contrast, the test has also been validated to identify patients with significant absolute risk reduction who benefit from RT and might be undertreated by BCS without RT. These patients might be good candidates for treatment with BCS plus RT, depending on the risk tolerance and other factors specific to the individual patient.

## Data availability statement

The datasets presented in this article are not readily available because proprietary biomarker and algorithmic data will remain confidential and will not be shared. Requests to access the datasets should be directed to TB, tbremer@preludedx.com.

## Ethics statement

The studies involving human participants were reviewed and approved by the Uppsala University Regional Ethical Review Board, University of Massachusetts Medical School Tissue and Tumor Bank Institutional Review Board, the Kaiser Permanente Northwest Institutional Review Board, Umeå University Ethics Review Committee, and the Royal Melbourne Hospital and Affiliated Hospitals Ethics Review Board. Written informed consent for participation was not required for this study in accordance with the national legislation and the institutional requirements.

## Author contributions

DD and TB contributed to conceptualization of the study. DD, TB, and KM provided methodology for the study procedures and data analysis. TB was involved in providing resources and project administration, while DD, CS, JS, and PW provided overall supervision of the study. DD, SH, JS, TB, and KM were responsible for data collection. TB provided funding acquisition. DD, SH, JS, KM, SS, and TB were responsible for data curation. KM, SS, and TB contributed to software needed to organize, curate, and analyze the study data, while TB provided formal analysis of the data and KM, SS and TB participated in validation of the data analysis. DD, JS, KM, CS, SS, and TB performed visualization of the data. DD, KM, and TB provided the original draft of the manuscript and CS, PW, SH, and SS participated in review and editing of the final manuscript. All authors contributed to the article and approved the submitted version.
