# Supplementary material for: Analytical validation of the 7-gene biosignature for prediction of recurrence risk and radiation therapy benefit for breast ductal carcinoma in situ
Source: Front Oncol. 2023 May 19;13:1069059. doi: 10.3389/fonc.2023.1069059 (PMC10236475; doi:10.3389/fonc.2023.1069059)
Supplement: Supplementary file 1 [file DataSheet_1.docx]

**Supplemental Table 1.** Protein expression for 7 genes in normal organ tissues*.

| **Organ tissue** | **PgR** | **HER2** | **Ki67** | **COX2** | **P16/INK4** | **SIAH2** | **FOXA1** |
| --- | --- | --- | --- | --- | --- | --- | --- |
| **Cerebrum  (n=6)** | Negative | Negative | Negative | Negative | Negative | Negative | Negative |
| **Cerebellum**  **(n=3)** | Negative | Negative | Negative | Negative | Negative | Negative | Negative |
| **Adrenal gland**  **(n=3)** | Negative | Negative | Negative | Positive | Negative | Negative | Negative |
| **Pancreas**  **(n=3)** | Negative | Negative | Negative | Positive | Negative | Negative | Negative |
| **Lymph node**  **(n=3)** | Negative | Negative | Positive | Positive | Negative | Positive | Negative |
| **Trachea**  **(n=3)** | Negative | Negative | Negative | Positive | Negative | Negative | Negative |
| **Testis**  **(n=3)** | Negative | Negative | Positive | Positive | Negative | Positive | Negative |
| **Thyroid gland**  **(n=3)** | Negative | Negative | Negative | Positive | Negative | Negative | Negative |
| **Spleen**  **(n=3)** | Negative | Negative | Positive | Positive | Negative | Positive | Negative |
| **Thymus gland**  **(n=3)** | Negative | Negative | Positive | Negative | Negative | Positive | Negative |
| **Bone marrow**  **(n=3)** | Negative | Negative | Negative | Negative | Negative | Positive | Negative |
| **Lung**  **(n=3)** | Negative | Negative | Positive | Positive | Negative | Negative | Negative |
| **Heart**  **(n=3)** | Negative | Negative | Negative | Negative | Negative | Negative | Negative |
| **Esophagus**  **(n=3)** | Negative | Negative | Positive | Positive | Negative | Positive | Negative |
| **Stomach**  **(n=3)** | Negative | Negative | Negative | Positive | Negative | Negative | Negative |
| **Small intestine**  **(n=3)** | Negative | Negative | Positive | Positive | Negative | Positive | Negative |
| **Colon**  **(n=3)** | Negative | Negative | Positive | Positive | Negative | Positive | Negative |
| **Liver**  **(3)** | Negative | Negative | Negative | Positive | Negative | Negative | Negative |
| **Kidney**  **(n=3)** | Negative | Negative | Negative | Positive | Negative | Negative | Negative |
| **Prostate**  **(n=3)** | Negative | Negative | Negative | Negative | Negative | Negative | Negative |
| **Uterus**  **(n=3)** | Positive | Negative | Negative | Negative | Negative | Negative | Negative |
| **Skeletal muscle**  **(n=3)** | Negative | Negative | Negative | Negative | Negative | Negative | Negative |
| **Skin**  **(n=3)** | Negative | Negative | Negative | Negative | Negative | Negative | Negative |
| **Nerve**  **(n=3)** | Negative | Negative | Negative | Negative | Negative | Negative | Negative |
| **Pericardium**  **(n=1)** | Negative | Negative | Negative | Positive | Negative | Positive | Negative |

* Normal tissues adjacent to tumors were excluded from the study.

## Supplemental Table 2. Expected positive and negative protein expression for 7 genes in normal organ tissue and carcinomas*.

| **Biomarker** | **Tissues with Expected Negative Expression (Tissue Format)** | **Tissues with Expected Positive Expression (Tissue Format)** |
| --- | --- | --- |
| **FOXA1** | Normal tissues including cerebrum, cerebellum, adrenal gland, ovary, spleen, thymus gland, bone marrow, lung tissue, cardiac muscle tissue, esophagus tissue, stomach, liver, kidney, endometrium, skeletal muscle, skin, nerve tissue, are not expected to have negative expression^1^  (FDA999w2 TMA from Biomax) | Colon adenocarcinoma and prostate adenocarcinoma are expected to have strong positive expression. (BC000119b, Biomax) |
| **SIAH2** | Normal cerebrum and adrenal gland tissue were expected to have negative expression.^2^ Normal cerebrum TMA was obtained from US Biomax  (GLN241a and FDA999w2 TMA). | Lung squamous cell carcinomas^3^ and colon adenocarcinomas^4^ are expected to have strong positive expression. Prostate adenocarcinoma neoplastic cells >1%^5^, Multiple organ cancer tissue array was obtained from US Biomax (BC000119b). Breast DCIS and invasive carcinoma^6^ (BR301TMA from Biomax) |
| **PgR** | Invasive breast carcinoma from external lab (BR1141a, BR1401 TMAs from US Biomax). | Invasive breast carcinoma from external lab (BR1141a, BR1401 TMAs from US Biomax) |
| **HER2** | Invasive breast carcinomas^7^ from external lab (BR1141a, BC08013d, BR1401 TMAs from US Biomax). | Invasive breast carcinomas from external lab (BR1141a, BC08013d, BR1401 TMAs from US Biomax). |
| **COX-2** | Normal Uterus^8^ (FDA999w2 TMA from US Biomax) and normal skin TMA (SKN1001 US Biomax). | Liver (LV805b TMA from US Biomax) DCIS and invasive breast carcinoma (BR301 TMA from US Biomax). |
| **Ki-67** | Invasive breast carcinomas (BR1141a, BC08013d, BR1401, BR301 TMAs from US Biomax). | Invasive breast carcinomas from external lab (BR1141a, BC08013d, BR1401, BR301TMA’s from US Biomax). |
| **p16** | Normal tissue including the cerebrum, cerebellum, adrenal gland, spleen, thymus gland, bone marrow, lung tissue, cardiac muscle tissue, esophagus tissue, stomach, liver, kidney, endometrium, skeletal muscle, skin, nerve tissue stained negatively for the p16. (FDA999w2 TMA from US Biomax). | HPV +ve squamous cell carcinomas (Cervix and Head and neck); Tissue sections with known p16 status were obtained from Bioptions. |

***** Normal adjacent tissues to tumors were excluded from the study

**Supplemental Table 3.** Number needed to treat (NNT) by risk group and performance summary statistics for a combined multinational cohort observational DCISionRT validation (n=926).

**A: DCISionRT Biosignature Low Risk Group vs Not Low Risk Group.**

| **RT Invasive IBR Risk Reduction** | | | |  | **RT Total IBR Risk Reduction** | | | |
| --- | --- | --- | --- | --- | --- | --- | --- | --- |
|  | **RT  Benefit** | **No RT Benefit** |  |  |  | **RT  Benefit** | **No RT Benefit** |  |
| **DCISionRT  not Low Risk** | $\hat{TP}$= 53 | $\hat{FP}$= 535 | PPV = 9% |  | **DCISionRT  no Low Risk** | $\hat{TP}$= 105 | $\hat{FP}$= 483 | PPV = 18% |
| **DCISionRT Low Risk** | $\hat{FN}$ = 4 | $\hat{TN}$= 334 | NPV = 99% |  | **DCISionRT Low Risk** | $\hat{FN}$= 4 | TN = 334 | NPV = 99% |
|  | Sensitivity 93% | Specificity 38% |  |  |  | Sensitivity 96% | Specificity 41% |  |

**B: EBCTCG DCIS RCT - Nuclear Grade 3 vs Nuclear Grade 1 or 2.**

| **RT Invasive IBR Risk Reduction** | | | |  | **RT Total IBR Risk Reduction** | | | |
| --- | --- | --- | --- | --- | --- | --- | --- | --- |
|  | **RT  Benefit** | **No RT Benefit** |  |  |  | **RT  Benefit** | **No RT Benefit** |  |
| **Nuclear Grade 3** |  | $\hat{FP}$= 592 | PPV = 7.5% |  | **Nuclear Grade 3** | $\hat{TP}$= 101 | $\hat{FP}$ = 539 | PPV = 16% |
| **Nuclear Grade 1 or 2** | $\hat{FN}$= 78 | $\hat{TN}$= 899 | NPV = 92% |  | **Nuclear Grade 1 or 2** | $\hat{FN}$ = 151 | $\hat{TN}$ = 826 | NPV = 85% |
|  | Sensitivity  38% | Specificity  60% |  |  |  | Sensitivity  40% | Specificity  61% |  |

|  | **RT Benefit** | **No RT Benefit** |  | **Summary Statistics** |
| --- | --- | --- | --- | --- |
| Test Positive for RT Benefit (X>c), N_POS_ | a. True Positive (TP) | b. False Positive (FP)  b=N_POS_-a |  | PPV = a/(a+b) =  a/N_POS_  N_POS_ = a+b |
| Test Negative for RT Benefit  (X≤c), N_NEG_ | c. False Negative (FN)  c = N_NEG_-d | d. True Negative (TN) |  | NPV = d/(c+d) =  d/N_NEG_  N_NEG_ = c+d |
|  |  |  |  |  |
| Summary Statistics | Sensitivity = a/(a+c) = TP/(TP+FN) | Specificity = d/(b+d) = TN/(TN+FP) |  |  |

**Supplemental Table 4.**

**Equation 3:** Confusion Matrix with Summary Statistics for count data. NPV – negative predictive value; PPV – positive predictive value; TP – true positive; FP – false positive; FN – false negative; TN – true negative; N_POS_ = Number of Test Positive; N_NEG_ = Number of Test Negative

**
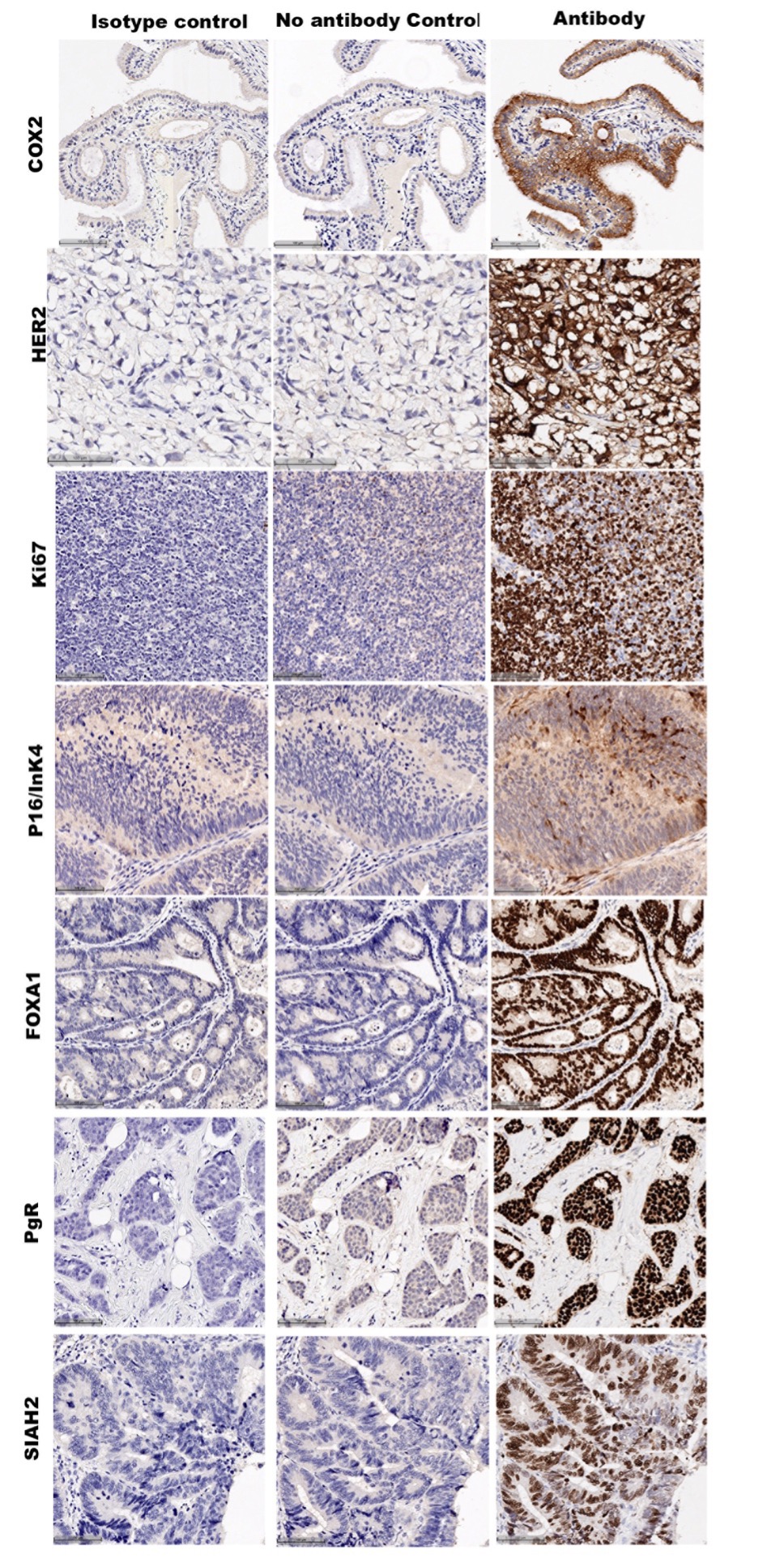
**

**Supplemental Figure 1 Representative Molecular Assay results for isotype and no-antibody controls.** Tissue type used for each antibody are: COX2- Normal Gall Bladder; HER2- Invasive Breast Carcinoma, KI67- Tonsil, P16/INK4A - Colon Adenocarcinoma, FOXA1- Colon Adenocarcinoma, PgR- Invasive Breast Carcinoma, SIAH2- Colon Adenocarcinoma.


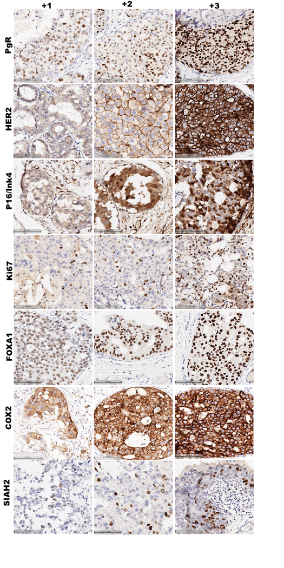


**Supplemental Figure 2** **Representative molecular assay results in DCIS tissue samples with varying levels of protein expression.**

**REFERENCES:**

1. Wang, L.L.*, et al.* The transcription factor FOXA1 induces epithelial ovarian cancer tumorigenesis and progression. *Tumour Biol* **39**, 1010428317706210 (2017).

2. <https://www.proteinatlas.org/ENSG00000181788-SIAH2/tissue>.

3. Moreno, P.*, et al.* The Expression of the Ubiquitin Ligase SIAH2 (Seven In Absentia Homolog 2) Is Increased in Human Lung Cancer. *PLoS One* **10**, e0143376 (2015).

4. Wang, D., Ma, L., Wang, B., Liu, J. & Wei, W. E3 ubiquitin ligases in cancer and implications for therapies. *Cancer Metastasis Rev* **36**, 683-702 (2017).

5. Qi, J.*, et al.* The E3 ubiquitin ligase Siah2 contributes to castration-resistant prostate cancer by regulation of androgen receptor transcriptional activity. *Cancer Cell* **23**, 332-346 (2013).

6. van der Willik, K.D.*, et al.* SIAH2 protein expression in breast cancer is inversely related with ER status and outcome to tamoxifen therapy. *Am J Cancer Res* **6**, 270-284 (2016).

7. Burstein, H.J. The distinctive nature of HER2-positive breast cancers. *N Engl J Med* **353**, 1652-1654 (2005).

8. St-Louis, I.*, et al.* Expression of COX-1 and COX-2 in the endometrium of cyclic, pregnant and in a model of pseudopregnant rats and their regulation by sex steroids. *Reprod Biol Endocrinol* **8**, 103 (2010).
